# Fissures of the Tongue

**Published:** 1891-03-07

**Authors:** 


### SURGERY.

### FISSURES OF THE TONGUE.

Fissures of the tongue not infrequently prove very intractable and difficult to cure. This, doubtless, is partly due to the constant movements of the tongue, and partly, too, to the presence and action of the saliva and food. We have found good results from the application of balsam of Peru, as in the following formula: Solution of cocaine, 10 per cent. ʒi; balsam of Peru, ʒii.; glycerine, ʒii. Dr. Schwimmer recommends a ten to twenty-per-cent. solution of papayotin (papain) in glycerine and water. He reports that, of twenty-five cases in which this solution was used, there was only one remained uncured. Many of the successful cases had been treated with applications of chromic acid, nitrate of silver, or iodoform without a favourable result. The fissures should be dried first before the application, which must be repeated five or six times daily. The internal administration of opium, combined with a simple carbolic acid mouth-wash locally, cured an intractable case under Mr. Shield.

